# Panoramic Magnetic Resonance Imaging of the Breast With a Wearable Coil Vest

**DOI:** 10.1097/RLI.0000000000000991

**Published:** 2023-05-27

**Authors:** Michael Obermann, Lena Nohava, Roberta Frass-Kriegl, Onisim Soanca, Jean-Christophe Ginefri, Jacques Felblinger, Paola Clauser, Pascal A.T. Baltzer, Elmar Laistler

**Affiliations:** From the High Field MR Center, Center for Medical Physics and Biomedical Engineering, Medical University of Vienna, Vienna, Austria (M.O., L.N., R.F.-K., O.S., E.L.); IADI, Université de Lorraine, Inserm, Nancy, France (L.N., J.F.); Laboratoire d'Imagerie Biomédicale Multimodale Paris Saclay (BioMaps), CEA, CNRS, Inserm, Université Paris-Saclay, Paris, France (J.-C.G.); and Department of Biomedical Imaging and Image-Guided Therapy, Division of General and Pediatric Radiology, Medical University of Vienna, Vienna, Austria (P.C., P.A.T.B.).

**Keywords:** magnetic resonance imaging, radiofrequency coil, flexibility, wearability, patient comfort, panoramic view, breast cancer, breast imaging, supine, prone

## Abstract

**Background:**

Breast cancer, the most common malignant cancer in women worldwide, is typically diagnosed by x-ray mammography, which is an unpleasant procedure, has low sensitivity in women with dense breasts, and involves ionizing radiation. Breast magnetic resonance imaging (MRI) is the most sensitive imaging modality and works without ionizing radiation, but is currently constrained to the prone imaging position due to suboptimal hardware, therefore hampering the clinical workflow.

**Objectives:**

The aim of this work is to improve image quality in breast MRI, to simplify the clinical workflow, shorten measurement time, and achieve consistency in breast shape with other procedures such as ultrasound, surgery, and radiation therapy.

**Materials and Methods:**

To this end, we propose “panoramic breast MRI”—an approach combining a wearable radiofrequency coil for 3 T breast MRI (the “BraCoil”), acquisition in the supine position, and a panoramic visualization of the images. We demonstrate the potential of panoramic breast MRI in a pilot study on 12 healthy volunteers and 1 patient, and compare it to the state of the art.

**Results:**

With the BraCoil, we demonstrate up to 3-fold signal-to-noise ratio compared with clinical standard coils and acceleration factors up to 6 × 4. Panoramic visualization of supine breast images reduces the number of slices to be viewed by a factor of 2–4.

**Conclusions:**

Panoramic breast MRI allows for high-quality diagnostic imaging and facilitated correlation to other diagnostic and interventional procedures. The developed wearable radiofrequency coil in combination with dedicated image processing has the potential to improve patient comfort while enabling more time-efficient breast MRI compared with clinical coils.

Breast cancer is the most commonly diagnosed malignant cancer and a leading cause of cancer-related death in women worldwide.^1^ Early detection of malignant lesions is considered one of the key factors for reducing breast cancer morbidity and mortality.

Digital x-ray mammography (DM) is the standard screening modality due to its procedural simplicity, accessibility, and proven effectiveness regarding patient outcomes.^2–4^ However, DM has low sensitivity in the substantial proportion of women with dense breast tissue^5^ and lacks functional information, making DM a suboptimal imaging modality for lesion characterization. As a result, approximately every second woman undergoes imaging, which is not well suited to her anatomy.^6^ X-ray–based screening is not recommended below the age of 45 years in most national screening programs^7^ due to the risks of ionizing radiation together with lower incidence rates and lower diagnostic performance due to a higher proportion of women with dense breasts. In addition, DM requires breast compression, causing discomfort or pain and can consequently reduce attendance rates.^8^

The limitations of x-ray–based breast imaging modalities can be alleviated by the use of magnetic resonance imaging (MRI) as it does not rely on ionizing radiation, yields excellent soft tissue contrast, and has the highest sensitivity for detection of breast cancer^9,10^—independent of breast density.^11^ Breast MRI examinations commonly involve the intravenous administration of gadolinium-based contrast agents to evaluate tumor vascularization. Contrast-enhanced breast MRI can reliably distinguish between benign and malignant breast lesions.^12,13^ In addition, the imaging method has been shown to be the superior assessment modality for early detection of breast cancer, both in women with high risk of breast cancer and very dense breasts,^14–17^ and was recently suggested for tailored screening.^9^

Typically, breast MRI is performed with the patient lying on her front (prone) with exposed breasts hanging into cup-shaped molds of a rigid radiofrequency (RF) coil. This current clinical setup does not exploit the full potential of breast MRI for the following reasons:

First, due to gravitational breast tissue deformation in the prone position, diagnosis and treatment are complicated since the breast shape differs considerably from the posture in supine ultrasound (US) or surgery,^18,19^ whereas acquisition in the supine position facilitates image fusion^20–22^ and tumor localization during US-guided biopsy, surgery, or radiation therapy.^23^ In addition, the prone posture is uncomfortable for the patient, and the lack of physical support of the hanging breasts may lead to motion artifacts, which compromise image quality.^24^ The feasibility of performing breast MRI in the supine position has been demonstrated.^18^ These arguments call for a new methodological approach enabling supine breast MRI.

Second, standard coils for (prone) breast MRI are designed to accommodate rather large breasts, which leads to reduced signal-to-noise ratio (SNR) for small breasts and precludes the measurement of very large breasts. Radiofrequency coils that can be form-fitted to the anatomical region of interest have been in the focus of recent developments. In particular, when the anatomical intersubject variability is high, as it is the case with the female breast, they can be better adapted to the subject's shape and thereby achieve higher SNR. Existing approaches and research prototypes range from rigid-adjustable^25–27^ to flexible,^28,29^ and even stretchable coils.^30–33^ A flexible coil vest prototype for supine breast MRI has recently been briefly presented,^34^ but not described or tested in detail. Coaxial transmission line resonators (“coaxial coils”) with 1 gap in the inner and outer conductor of a coaxial cable were already described in 1946^35^ and the idea to use them as flexible receive coils for MRI dates back to Zabel et al.^36^ Based on several recent studies^37–39^ characterizing coaxial coils, the potential of this technology for flexible coil design has been reconsidered by the community.^40–42^ Radiofrequency coils suitable for supine breast MRI are either semiflexible (eg, “Body 18,” Siemens Healthineers, Erlangen, Germany) or fully flexible coils (eg, “AIR™ coils,” GE Healthcare, Waukesha, WI or “Contour coils,” Siemens Healthineers, Erlangen, Germany). Although some of these coils allow for proof-of-concept supine breast MRI, they are not optimized for breast MRI leading to low SNR, potentially unwanted aliasing artifacts and practical restrictions. Therefore, a flexible coil for supine breast MRI, overcoming the limitations of existing research prototypes and product coils, is needed.

Third, prone breast images are conventionally displayed upside down in an axial Cartesian view. However, for supine imaging, this view is suboptimal, since the breast tissue is flattened and distributed over the chest wall making the axial cross-section of the breast considerably thinner. A coronal view may be more suited for supine images, but is still not optimal for viewing the lateral parts of the breasts. In both cases, the high number of slices leads to long reading times. Consequently, a new visualization paradigm for supine breast MRI is required.

All these issues underline the necessity of technical advancements in dedicated hardware and image processing for supine breast MRI. In this work we, therefore, propose “panoramic breast MRI,” which stands for the combination of a wearable RF coil, supine acquisition, and a panoramic visualization of breast MR images. We present and characterize the “BraCoil,” a flexible, vest-like receive-only coil array made of coaxial coil elements for 3 T MRI that can be used both in supine and prone positioning. The coil was developed for 3 T MRI, since the higher achievable SNR compared with lower field strength allows for acquisitions with higher in-plane resolution and/or thinner slices, which can improve the detection of small lesions in the breast^43,44^ and has benefits, especially for imaging techniques such as DWI.^45^ The BraCoil is designed to strongly improve comfort, enable consistency in the breast shape with other imaging and therapeutic modalities, reduce preparation and acquisition time, and increase SNR. In addition, we introduce a panoramic visualization of both breasts similar to dental panoramic x-ray, overcoming the limitations of the Cartesian view in supine breast MRI. We validate the panoramic breast MRI approach in a healthy volunteer study against 2 commercial coils and demonstrate the fundamental aptitude of this technique for clinical assessment in an exemplary case of a breast cancer patient.

## MATERIALS AND METHODS

### Coil Development

A flexible breast coil array that can be worn like a sports bra was conceived and implemented. The shape of the textile parts of the vest and the matching coil array layout were developed based on prior consultation of experienced breast radiologists and population statistics^46,47^ to accommodate a large range of body shapes and breast sizes while sufficiently covering both breasts and allowing for assessment of the axillary lymph nodes.

**FIGURE 1:**
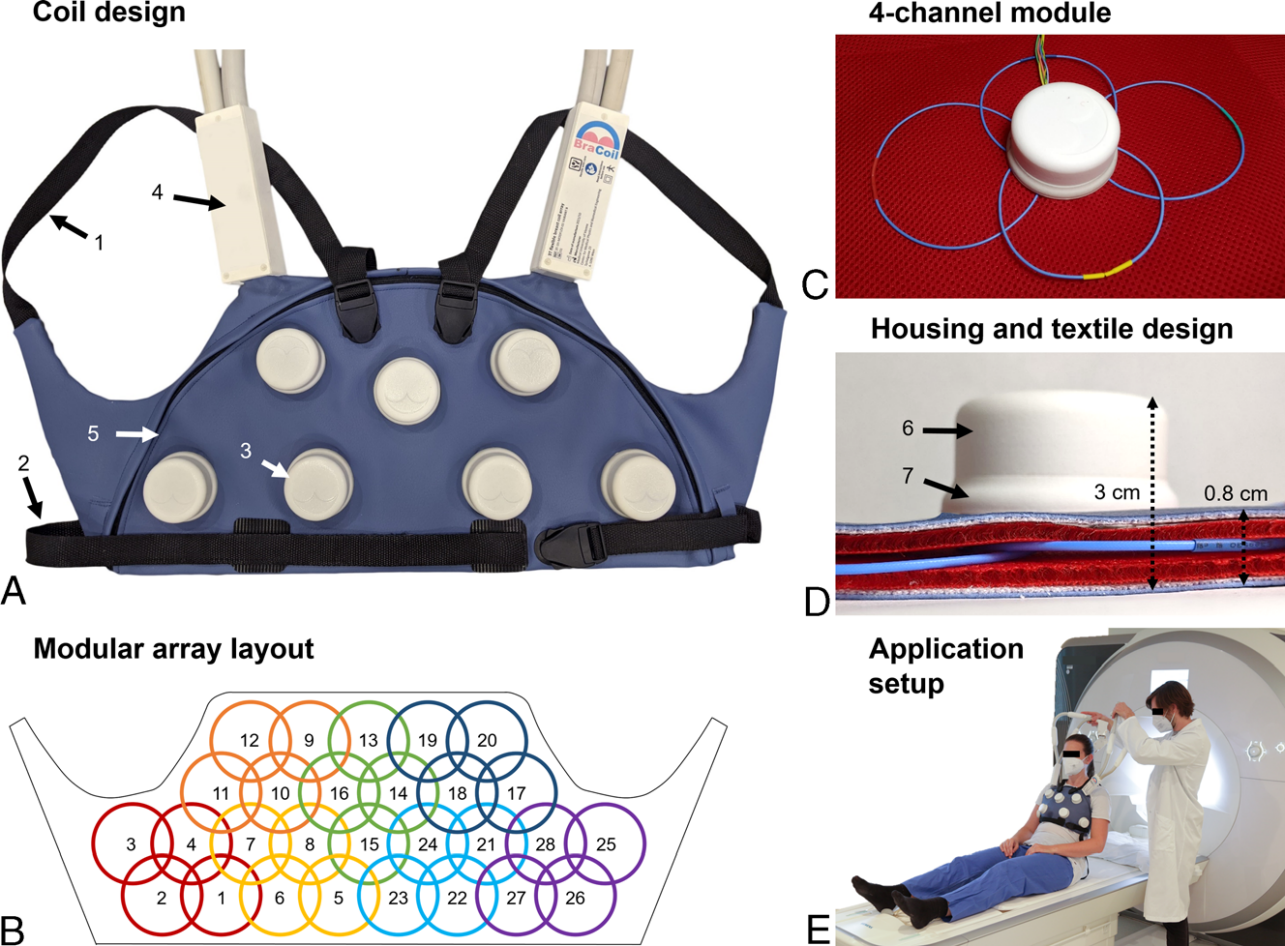
Design features of the “BraCoil.” The relevant coil design features are summarized, showing the shape and arrangement of the coil array, its modules and elements, as well as the proposed measurement setup. A, The coil vest is ergonomically shaped and adjustable to the subject's anatomy with shoulder and waist belts (arrows 1, 2). Electrical components are enclosed by multilayer textile and 3D-printed housings. Coil elements are grouped into modules, each with its own interface housing (3). Shoulder interface boxes (4) contain cable traps for common mode current suppression and cable strain relief toward coils and system plugs. A semicircular plastic zipper (5) allows easy access for maintenance. B, 28 elements are organized in seven 4-channel modules, each wired with 4 receive cables and a twisted cable pair for module-wise switching of the detuning circuits. C, A 4-channel module showing the coaxial coil elements, housing, and the bundle of receive cables and bias lines. The outer gaps in the coax shield are covered with heat shrink tubes. D, Coil elements are protected by 2 cushion layers (red) and overall enclosed by medical grade synthetic leather. Cabling between modules and shoulder interfaces is running between the top cushion layer and the synthetic leather. The housing for each module consists of a flat bottom part, a slidable ring (6) and a cap (7). The contained electronics are detailed in the methods section. E, The procedure of coil positioning on the subject is shown.

The resulting design (Fig. 1A) is a flexible 28-channel receive-only coil array (Fig. 1B), organized in seven 4-channel modules (Fig. 1C) and enclosed by 3D-printed interface housings and textile layers (Fig. 1D). The coil array has an overall size of 55 × 25 cm^2^ with the sensitive imaging area exceeding the array size at all sides. Each module contains 4 circular, single-gap coaxial coils of 8 cm diameter made from thin and very flexible coaxial cable with an interruption (gap) in both inner and outer conductor to achieve self-resonance close to the Larmor frequency of 123.2 MHz for a 3 T MR scanner (Prisma Fit, Siemens Healthineers, Erlangen, Germany). The electrical circuit schematic for a single coaxial coil's interfacing to the MR scanner is sketched in the supplementary material (see Figure, Supplemental Digital Content 1, http://links.lww.com/RLI/A817). In contrast to another design,^34^ the BraCoil's layout is confined to expected regions of breast tissue, and thereby avoids potential fold-over artifacts from the abdomen and unnecessary hardware and weight.

The vest can be adjusted to the subject's contours with waist and shoulder belts to ensure a tight fit and optimize the achievable SNR. Thanks to the small weight of ≈1.5 kg distributed on the subject (excluding system cables, since they are supported by the patient bed), the array can be worn comfortably in supine position (Fig. 1E). Image acquisition in prone position is also possible using an additional support plate placed on the patient bed. Details on the RF coil interfacing, module design, and array assembly can be found in the supplementary material (Text, Supplemental Digital Content 2, http://links.lww.com/RLI/A818). Bench and phantom MR tests for coil characterization and patient safety testing were performed before volunteer and patient measurements (Text, Supplemental Digital Content 3, http://links.lww.com/RLI/A819).

### Study Design

In vivo MR measurements in this prospective controlled crossover study were conducted after approval of the Institutional Review Board (ethics committee, Medical University of Vienna, number 2137/2021) following the Declaration of Helsinki of 1975, and volunteers as well as the patient included in this study declared informed consent. The clinical investigation according to Regulation (EU) 2017/745 Article 62 was approved by the Austrian Federal Office for Safety in Health Care. The BraCoil is an investigational device and has neither FDA approval nor CE marking.

The study population consisted of 12 healthy female volunteers with mean body mass index 23 ± 4 (minimum, 17; maximum, 32) kg/m^2^, age 31 ± 5 (minimum, 25; maximum, 43) years, and bra sizes ranging from 70A to 90D, which corresponds to breast volumes ranging from 495 mL to 3020 mL. Bra sizes are indicated according to the Joint European standard for size labeling of clothes (EN13402). The sample size was chosen on the basis of a desired measurable difference by a factor of 1.2 between the BraCoil and reference coil's SNR (with an assumed standard deviation of 20%, resulting in an effect size of 1). Using a power of 0.8 and significance level α of 0.05 together with an estimated 20% dropout rate, a sample size of 10 was set as the minimum requirement for our study. The prospectively defined inclusion criteria for volunteers were female sex, age between 19 and 80 years, and a breast size between 70A and 90D, which can be considered representative for a larger population.^46,47^ To demonstrate the suitability of the BraCoil for breast cancer assessment, a 26-year-old female patient with a suspected breast lesion assigned BI-RADS 4, body mass index of 26 kg/m^2^, and bra size of 85E (breast volume, 2373 mL) was included in this study.

Healthy volunteers were measured with the BraCoil and 2 reference product coils in a chronologically randomized order: a dedicated breast coil “16-channel Sentinelle Breast Coil” (reference coil Ref1) and a semiflexible 18-channel multipurpose coil “Body 18” (reference coil Ref2) (both Siemens Healthineers, Erlangen, Germany). The patient was measured with the BraCoil and reference coil Ref1. One measurement session was performed per volunteer/patient and per coil.

To assess the preparation times required with the BraCoil in comparison to Ref1, an operator experienced with both coils repeated the coil setup on the patient table and patient positioning 5 times. The same experiment was performed with 2 experienced operators working together. In addition, an inexperienced user only familiar with the use of head coils performed the coil setup and positioning 3 times to evaluate the learning curve.

Prospectively selected end points for which MR data were collected were mean SNR and SNR homogeneity in the breast volume. With parallel imaging, the SNR is decreased by the square root of the acceleration factor *R*, and by the so-called geometry-factor (or short “*g*-factor”^48^), which is dependent on the correlation of signals between the individual coil channels and is spatially variable. Thus, another end point of this study were *g*-factor maps in a coronal slice with acceleration in left-right and head-foot direction. The number of slices containing breast tissue with and without panoramic reconstruction was evaluated.

The overall purpose of this study was to evaluate the BraCoil's MR and bench performance and combine novel hardware with efficient panoramic reconstruction methods. Prespecified research hypotheses or objectives were as follows: equal or increased mean SNR and SNR homogeneity in the breast volume compared with clinical standard reference products, high acceleration possibilities (ie, >3 × 2 for *g*-factors <2) due to the high channel count of the BraCoil, and a reduction of the overall number of slices to read after panoramic reconstruction as compared with a Cartesian axial or coronal view.

### In Vivo MRI Protocol

Magnetic resonance imaging scans were acquired on a 3 T MR scanner (Prisma Fit; Siemens Healthineers, Erlangen, Germany). The BraCoil's performance was compared with reference coils Ref1 and Ref2. Two subject positioning variants—supine and prone—were tested with the BraCoil and Ref2. For the measurements in prone position with the BraCoil, a simple plate with holes on the patient bed was used to accommodate the module housings. Ref1 only allows for imaging with the subject in prone position. All supine measurements were acquired with volunteers and a compliant patient briefed to perform abdominal breathing rather than chest breathing to limit motion artifacts.

Magnetic resonance sequence parameters used for the technical coil performance evaluation conducted with the healthy volunteer group and the multiparametric clinical protocol, which was tested on volunteers and finally used for the patient measurement are listed in the supplementary material (Table, Supplemental Digital Content 4, http://links.lww.com/RLI/A820).

The technical protocol included a T1-weighted fast low angle shot (FLASH^49^) acquisition to assess the coils' sensitive area. For SNR evaluation, a T1-weighted 3D gradient echo (GRE) sequence with low contrast between fat and water was chosen. To this end, the flip angle was adapted so that the MR signals originating from fat and water were similar in amplitude. For *g*-factor calculation T1-weighted 2D GRE scans of a few relevant coronal slices through the breast were acquired with the BraCoil in prone subject position. A noise-only scan (without RF transmit pulse) was acquired to obtain the noise correlation matrix used for SNR calculations.

The clinical protocol consisted of a high resolution and a fast dynamic T1-weighted 3D FLASH acquisition with spectrally attenuated inversion recovery (SPAIR) fat suppression, a T2-weighted turbo spin echo scan and a diffusion-weighted imaging (DWI) RESOLVE^50^ sequence. Volunteer measurements with reference coil Ref1 in prone position were performed using a similar custom protocol. The patient was also measured with the clinical BraCoil protocol set up for this study (supine) and a standard clinical protocol using Ref1 (prone). During the patient examination, dynamic contrast-enhanced T1-weighted imaging with fat suppression was performed. The gadolinium-based contrast agent used was 0.1 mmol/kg body weight of Dotarem (Guerbet, Villepinte, France). While with the BraCoil, 1 native and 7 fast T1-weighted scans were acquired within approximately 5.5 minutes (pause for contrast agent administration excluded); with Ref1, 1 native and only 4 postcontrast T1-weighted scans were acquired within the same scan time.

### Statistical Analysis and Data Postprocessing

Three-dimensional SNR maps for a nonaccelerated acquisition, that is, fully encoded k-space matrix, were calculated offline in MATLAB 2021b (The Mathworks, Inc, Natick, MA) from GRE imaging data and noise-only scans using the pseudo multiple replica method.^51^ Further, noise-only data were used to calculate the normalized noise correlation matrix for the smallest (bra size, 70A; breast volume, 495 mL), a medium-sized (bra size, 85B; breast volume, 1353 mL), and the largest (bra size, 90D; breast volume, 3020 mL) subject (see Figure, Supplemental Digital Content 5, http://links.lww.com/RLI/A821).

The mean 3D SNR was evaluated in a manually segmented region of interest (ROI) covering the entire breast tissue for all 5 measured setups (BraCoil and Ref2 supine and prone, Ref1 prone) using open-source image processing software (3D slicer v4.11^52–54^). The mean 3D SNR achieved with the BraCoil and the reference coils was compared using single-sided paired *t* tests.

Relative SNR homogeneity was assessed as the standard deviation divided by the mean SNR over the segmented breast volumes. The results for all subjects were compared between the different coil and positioning setups by single-sided paired *t* tests; the mean and standard deviation were calculated for each setup (as shown in Figure, Supplemental Digital Content 6, http://links.lww.com/RLI/A822).

To investigate the BraCoil's parallel imaging performance, we additionally calculated 2D SNR and *g*-factor maps with simulated acceleration factors of *R* = 2, 4, 6, and 8 in left-right (LR) and head-foot (HF) direction using open source Matlab code^55,56^ also based on the pseudo multiple replica method. Resulting *g*-factor maps were plotted in a coronal slice for 3 differently sized breasts.

**FIGURE 2:**
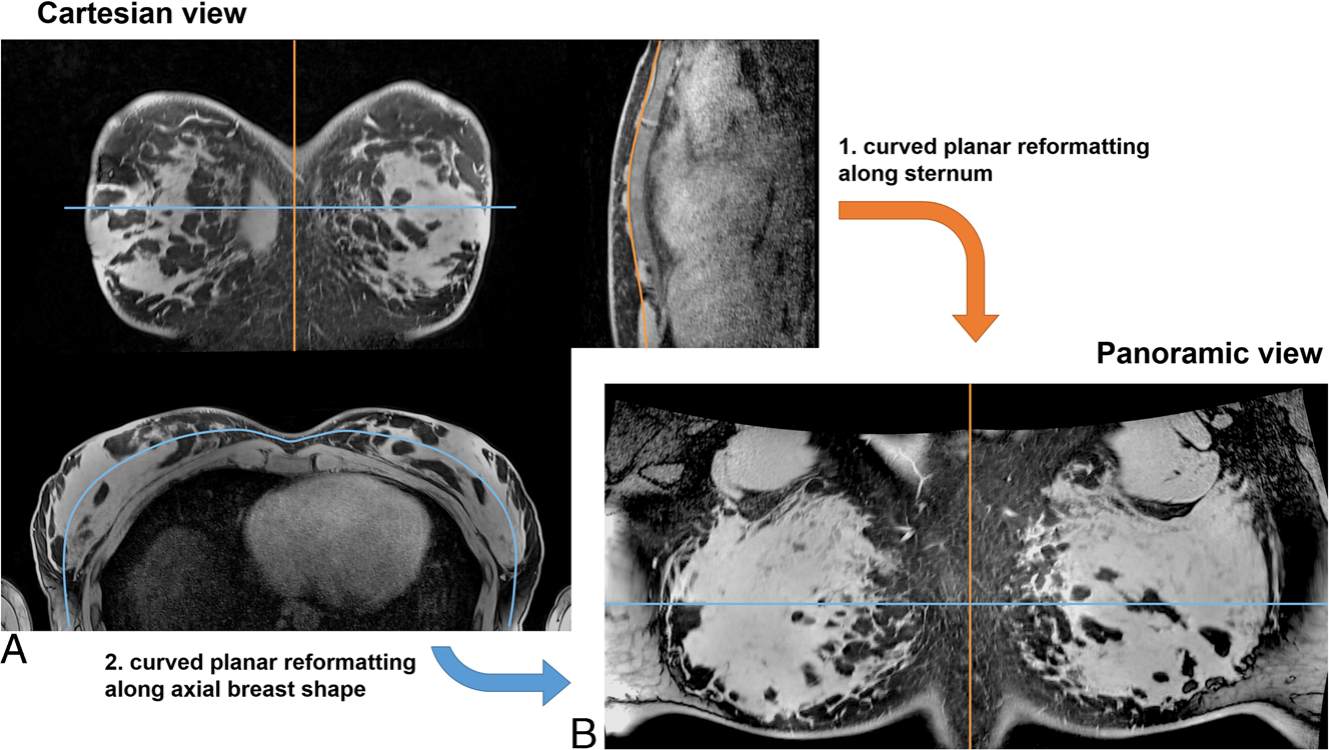
The panoramic breast view. To intuitively visualize both breasts in an efficient manner, a panoramic reconstruction comparable to panoramic dental x-ray was implemented. A, Two lines are manually defined in the Cartesian view: (1) along the sternum (orange) in the sagittal Cartesian view and (2) following the shape of the breast in the axial Cartesian view. The sagittal slice is positioned through the center of the sternum; the axial slice is chosen at the position of the fullest part of the breast. B, The panoramic view is created by consecutive curved planar reformatting along the 2 defined lines.

Since the breast tissue is distributed along the curved shape of the thorax when lying in the supine position, we hypothesize that displaying the images along the shape of the thorax rather than in Cartesian axial or coronal slice direction could be beneficial. We expect that such a “panoramic” visualization would lead to more breast tissue visible in each slice, thereby reducing the number of slices containing breast tissue. In addition, we believe that the panoramic view could be an intuitive new approach for assessing the anatomy and relative position of image features. Therefore, panoramic breast images were created by using 2 consecutive curved planar reformatting transforms along the sternum and along the breast shape in the axial view, respectively (see Fig. 2). The following workflow was used in 3D Slicer^52–54^ to reconstruct panoramic breast images:

1. Load T1-weighted, T2-weighted, and DWI images.
2. Display T1-weighted images (Fig. 2A).
3. Perform a linear transform T_rot,1_ rotating the image volume to maximize left-right symmetry
4. In the central sagittal slice through the sternum, manually draw a spline curve C_sag_ (orange line in Fig. 2) after the sternum.
5. Calculate the first curved planar reformatting transform T_sag_ according to C_sag_ flattening the volume in head-foot direction.
6. On the axial slice through the fullest part of the breast, manually draw a spline curve C_ax_ (blue line in Fig. 2). To minimize geometric distortion, this line should be drawn through the breast tissue with approximately equal distance between chest wall and body surface.
7. Calculate the second curved planar reformatting transform T_ax_ according to C_ax_ flattening the volume in left-right direction.
8. In the coronal view of the flattened volumes, perform another linear transform T_rot,2_ to optimize symmetry.
9. Apply all transforms to all loaded datasets and export images (Fig. 2B).

Panoramas of volunteer data with small, medium, and large breast volume (657/1338/3020 mL) and the patient data were created according to the workflow above.

To quantify the effect of the panoramic breast view, the total number of image slices comprising breast tissue was compared between supine BraCoil acquisitions in panoramic/axial/coronal view and prone acquisitions with Ref1 in axial view. To this end, the breast tissue segmentations used for SNR calculation were transformed into the panoramic view. The volumes were then tightly cropped to the extent of the breast tissue with more than 10 breast tissue voxels. The sizes of the remaining volumes in HF and AP direction were measured, and the relative size reductions of the flattened volume to the references were calculated.

## RESULTS

### Coil Performance

The scattering (S-) parameter and the normalized noise correlation matrix were measured and are displayed in the supplementary material (Figure, Supplemental Digital Content 5, http://links.lww.com/RLI/A821). From S-parameter measurements, coil impedance matching to 50 Ω and coupling between channels were determined for the smallest, the medium sized, and the largest subject within the cohort. Globally, matching (S_ii_) was better than −10 dB for all channels and subjects. Averaged over all channels, matching was −18.6 dB/−18.5 dB/−16.9 dB for the smallest, the medium, and the largest subject, respectively. The corresponding interelement coupling (S_ij_) was below −12.4 dB/−11.7 dB/−12.3 dB. The normalized noise correlation was between 6% and 7% on average. These bench values indicate proper implementation and functioning of the coil array.

In volunteer MR measurements, the investigated performance parameters were coverage, SNR average and homogeneity in the breast volume, and parallel imaging performance in terms of *g*-factors.

**FIGURE 3:**
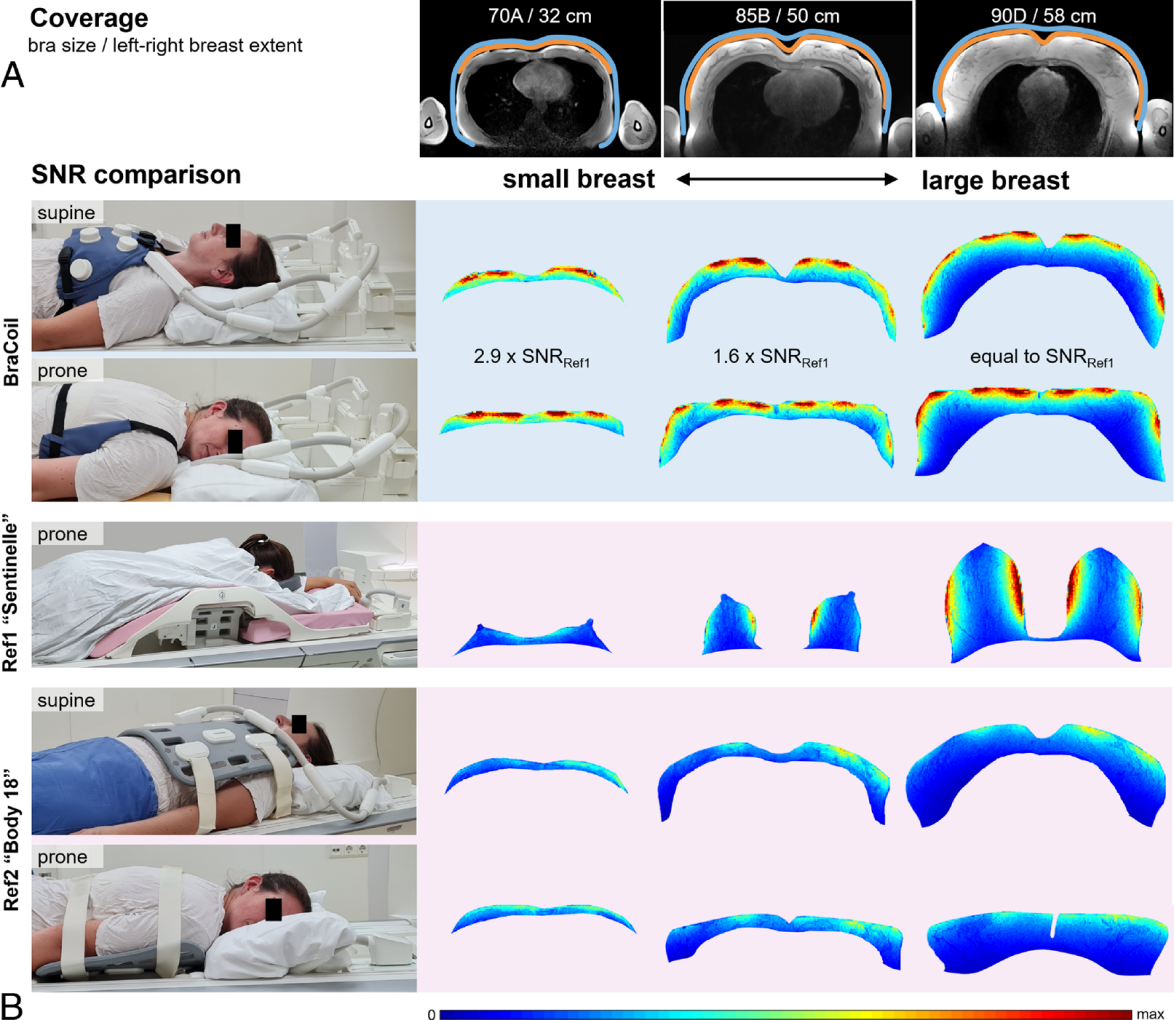
Performance of the BraCoil for different breast sizes and comparison to the clinical standard reference coils. The spatial distribution of SNR achieved with the BraCoil and the 2 reference coils is displayed, demonstrating that the BraCoil achieves higher SNR, particularly for smaller breasts. The flexibility of the coil ensures a tight fit and sufficient coverage for small and large breasts alike. A, Axial T1-weighted gradient echo images show the sensitive area (coverage) of the coil. The blue curves indicate the approximate position of the coil, orange curves indicate the left-right extent of the breasts measured along the body surface. Bra sizes and left-right extent of the breasts are indicated. B, SNR maps are shown for the BraCoil in supine and prone positioning of the patient, in prone position for reference coil Ref1 “16-channel Sentinelle Breast Coil,” and in supine and prone position for reference coil Ref2 “Body 18.” SNR was evaluated in the whole breast volume and nonbreast tissue was removed for display purposes.

As can be seen in Figure 3A, all sizes from the smallest to the largest breasts in the cohort of 12 healthy volunteers can be covered: for small breasts, the coil array wraps around the torso toward the back, whereas for large breasts, the sensitive area is still sufficient to cover the whole breast. The maximum left-right extent of the breast tissue strictly following the body surface was determined to be between 32 and 58 cm for the subjects investigated. The coil coverage is sufficient for left-right extents of the breasts of approximately 70 cm (corresponding to a bra size of ≈ 90E/95D/100C) and the approximate sensitive imaging area in head-foot direction is 30 cm.

**FIGURE 4:**
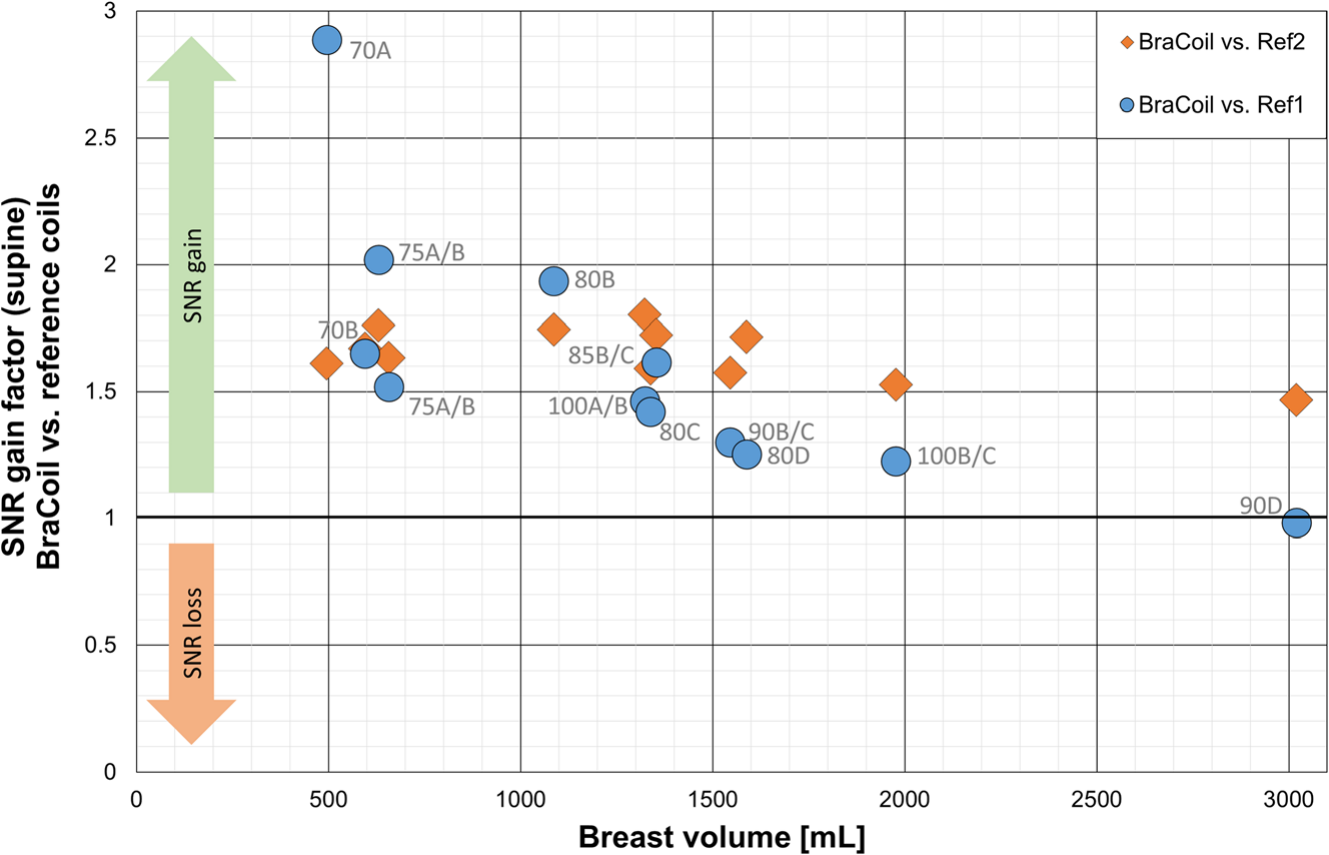
SNR gain factor of the BraCoil over reference coils in dependence on breast volume. The BraCoil achieves statistically significantly higher SNR than both reference coils, with a particular advantage for smaller breasts. The SNR gain factor SNR_BraCoil, ROI_/SNR_Ref, ROI_ of the BraCoil over the reference coils Ref1 “16-channel Sentinelle Breast Coil” (blue circles) and Ref2 “Body 18” (orange diamonds) within the breast region of interest (ROI) is plotted over the total breast volume for all subjects.

The results of the SNR comparison are shown in Figure 3B and Figure 4. Overall, in BraCoil measurements, the SNR was significantly improved compared with reference coils (*P* = 0.00005 for Ref1, *P* = 0.0000004 for Ref2), with a particular benefit for smaller breasts. The SNR gain over the standard breast coil Ref1 ranges from almost 3-fold SNR in the smallest breasts to equal SNR in the largest breasts. The SNR gain over the semiflexible coil Ref2 is roughly constant with an average of ≈1.7, that is, +70%.

Because of the nature of surface coils, the BraCoil's SNR rapidly decreases with distance to the coil elements; therefore, the relative SNR homogeneity within the breast volume was compared with the reference coils for all subjects of the study cohort. The homogeneity of the BraCoil is not significantly different from the clinical standard coil Ref1 (Figure, Supplemental Digital Content 6, http://links.lww.com/RLI/A822).

**FIGURE 5:**
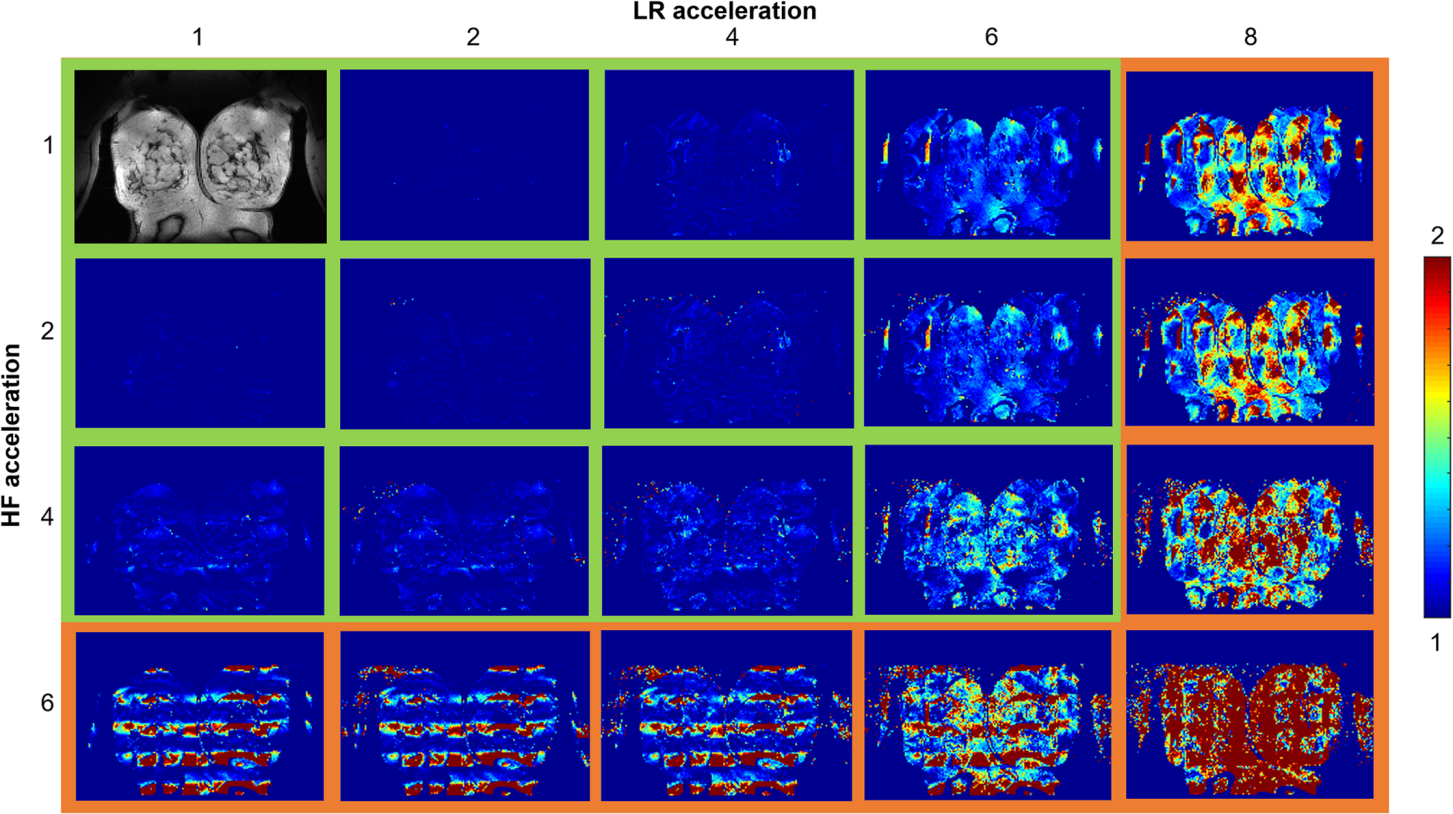
Parallel imaging performance. The BraCoil can be used for highly accelerated imaging. The distribution of the *g*-factor in a coronal slice (subject with medium breast size: volume, 1353 mL; bra size, 85B), acquired with the BraCoil in prone position, is shown. The maximum reasonably usable acceleration (*g* < 2) is 6 in LR direction, and 4 in HF direction. Maps are shown up to the point of failure at *R*_LR_ = 8 and *R*_HF_ = 6.

The high SNR and high channel count of the BraCoil can be used to accelerate image acquisition by using parallel imaging techniques. Maps of *g*-factor distribution for the BraCoil calculated for accelerations up to 8 in left-right (LR) and 6 in head-foot (HF) direction are shown in Figure 5 for a subject with medium breast size. Maps for other breast sizes can be found in the supplementary material (Figure, Supplemental Digital Content 7, http://links.lww.com/RLI/A823). The maximum acceleration factor with *g*-factors <2, which can be considered reasonable, was determined as 6 × 4 = 24. The higher value in LR direction is in agreement with the higher channel count of the coil array in that direction.

The results of the evaluation of preparation times are summarized in the supplemental material (Table, Supplemental Digital Content 8, http://links.lww.com/RLI/A824). For experienced operators, preparation times with the BraCoil are approximately 1 minute shorter than with Ref1. An inexperienced operator achieved the same preparation time in the third run of setting up the BraCoil. In addition, coil setup is easily achievable with only 1 operator with the BraCoil, whereas Ref1 may require 2 operators to lift the heavy and large coil onto the patient table.

### Panoramic Visualization

**FIGURE 6:**
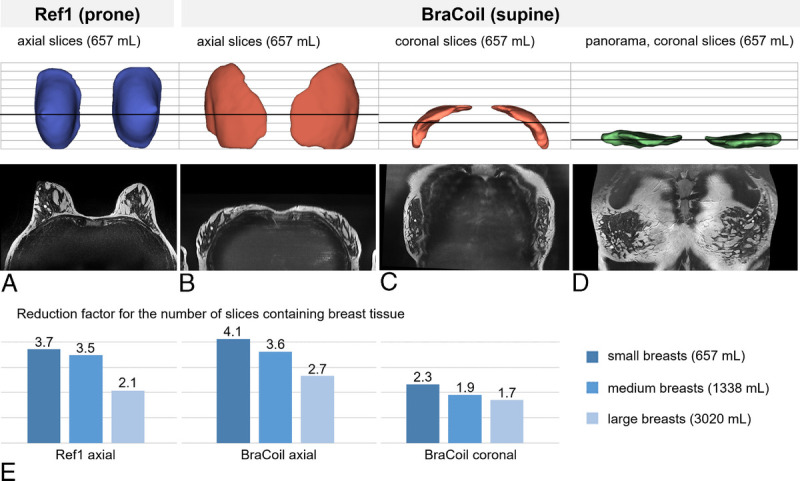
Evaluation of the efficiency of panoramic breast visualization in 3 subjects with a range of breast volumes. The panoramic approach reduces the amount of images that has to be evaluated by the radiologist. Exemplarily, a 3D-rendering of the segmented breast tissue (top row) and T2-weighted images (center row) from the subject with 657 mL breast volume is shown. The slice positions for the T2-weighted images are indicated by a black line through the breast segmentations: (A) “16-channel Sentinelle Breast Coil” (Ref1) prone data and (B) Bracoil supine data, both sliced axially. Bracoil supine data sliced coronally (C) on original volume, and (D) flattened volume after the panoramic transformation. In E, the reduction factor in the number of slices with panoramic (D) compared with Cartesian (A, B, C) visualization is plotted for small, medium, and large breasts.

Figures 6A to D show the segmented breast tissue from a subject with small breast volume (657 mL) and illustrate how slice numbers were evaluated for different imaging setups and views. Compared with axial images with either Ref1 in prone (Fig. 6A) or the BraCoil in supine position (Fig. 6B), the coronal acquisition with the BraCoil (Fig. 6C) already reduces the total number of slices containing breast tissue. By flattening the tissue using the panoramic visualization approach (Fig. 6D), the total number of slices decreases further. As shown in Figure 6E, depending on the breast size, the number of slices to be read was considerably reduced using panoramic breast MRI compared with the clinical breast coil and the BraCoil in Cartesian view: Supine BraCoil images with panoramic reformation decreased the total number of slices containing breast tissue by a factor of 2.1 to 3.7 compared with axial images acquired with Ref1 in prone position. A 2.7 to 4.1 (1.7 to 2.3)-fold reduction was obtained in comparison to the axial (coronal) view of supine BraCoil images. Consistently, the effect is higher for smaller breasts. This demonstrates that panoramic visualization can reduce the radiologists' effort by reducing the number of images they have to evaluate.

### In Vivo Panoramic Breast Imaging

**FIGURE 7:**
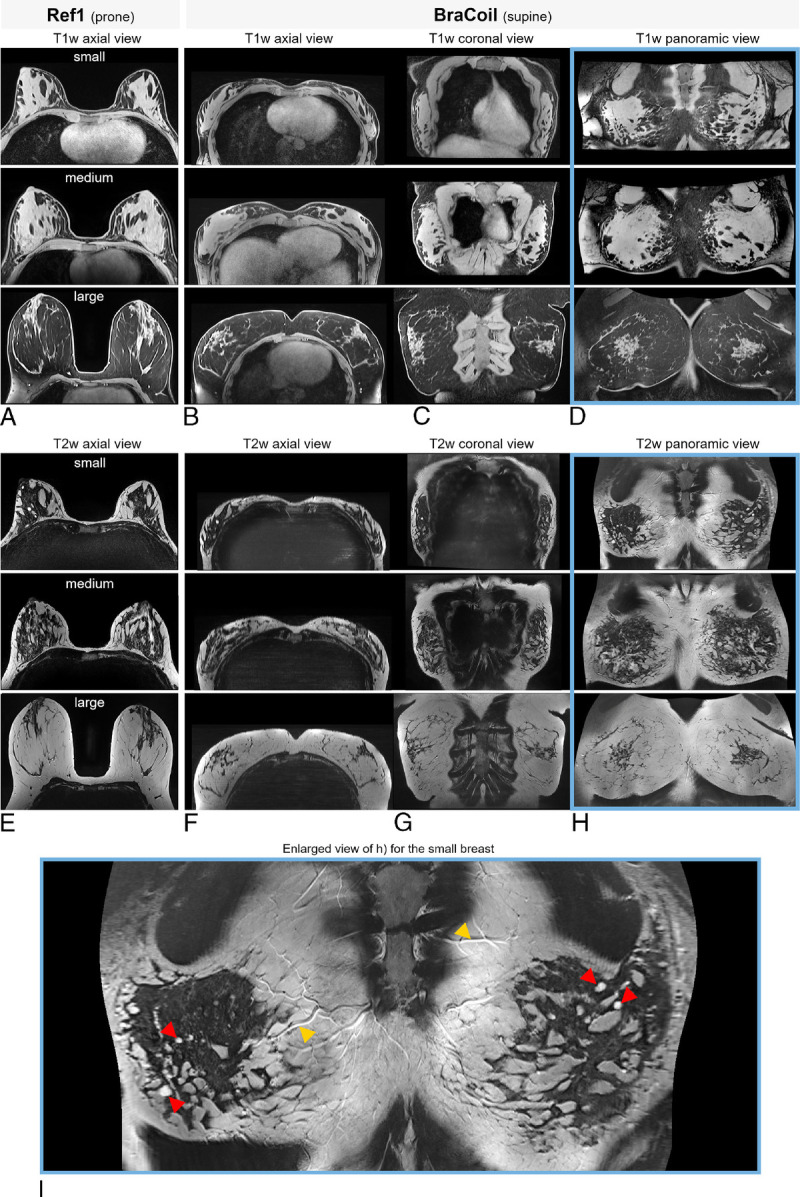
T1- and T2-weighted images from healthy volunteers. The potential of panoramic breast MRI is demonstrated on healthy volunteers. Excellent image quality can be observed throughout the data, and the intuitive nature of the panoramic view can be appreciated. T1-weighted (A–D) and T2-weighted (E–I) images are shown for subjects with small (657 mL), medium-sized (1338 mL), and large (3020 mL) breasts. Panoramic breast images are highlighted by blue borders. The left column (A, E) shows data acquired with Ref1 “16-channel Sentinelle Breast Coil” in the prone position. The other columns (B–D, F–I) show supine BraCoil data. For comparison to the reference images, in the second column (B, F), the axial view of the supine MRI data is shown. Column 3 displays the same data in a coronal view (C, G). In column 4, the proposed panoramic view of supine breast MRI data is presented. Panel I shows an enlarged version of H for the small breast to demonstrate the image quality. Red arrows, benign uncomplicated cysts; yellow arrows, vessels.

Magnetic resonance imaging data obtained with the BraCoil and Ref1 from 3 healthy volunteers representing the spectrum of investigated breast sizes are shown in Figure 7. The figure depicts rather small breasts (657 mL) with several benign cysts, medium-sized dense breasts (1338 mL), and large breasts (3020 mL) with high fat fraction. T1-weighted and T2-weighted supine BraCoil images acquired with free breathing volunteers and reconstructed using the panoramic view are of diagnostic quality. Fine anatomical structures including the distribution and amount of fibroglandular tissue are clearly distinguishable.

**FIGURE 8:**
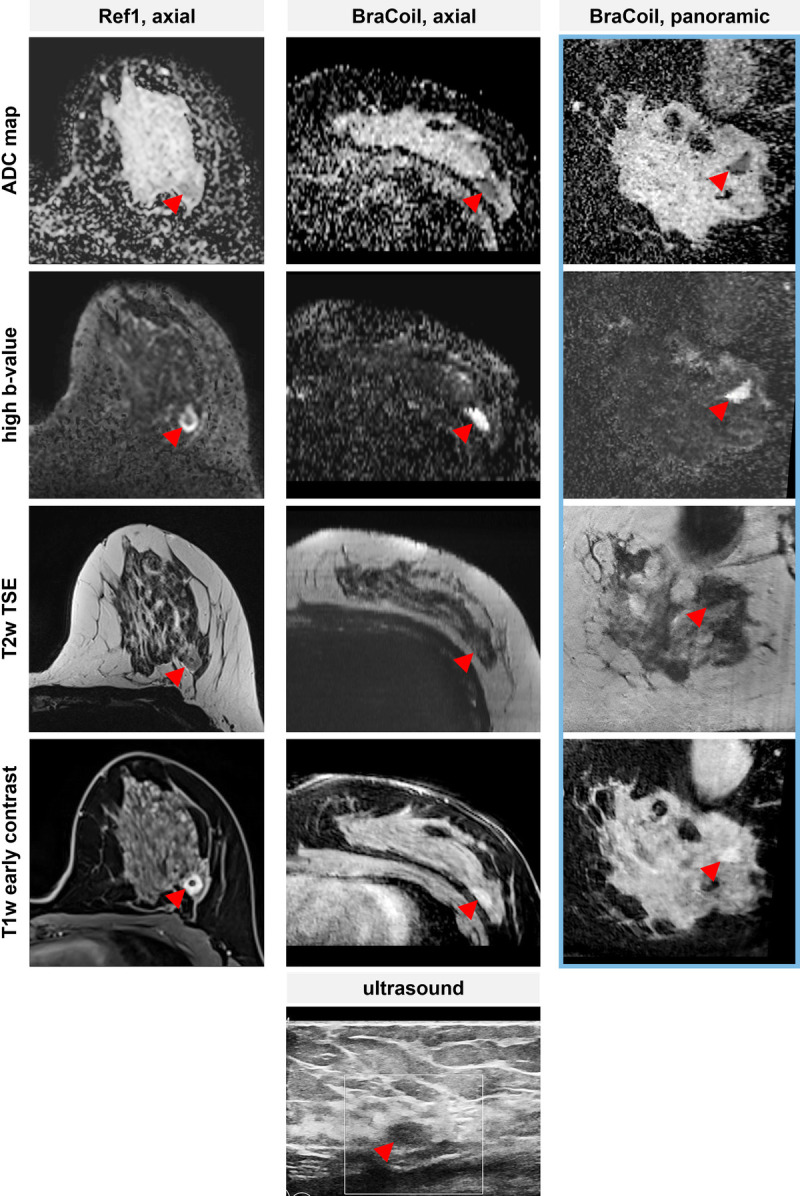
Clinical assessment case using the BraCoil in supine position. An exemplary case of clinical breast cancer assessment is presented. It highlights the advantages of supine imaging with the BraCoil. Because of the matching breast geometry between the panoramic breast MRI with the BraCoil and US, the lesion could be identified and biopsied in second-look US. The apparent diffusion coefficient (ADC) map (first row) shows low values of 1.18 × 10–6 mm^2^/s in the mass lesion at 2:00 in the left breast. Calculated high b-value (b = 1400 s/mm^2^) images (second row) show a corresponding hyperintensity of the lesion, while appearing slightly hyperintense on the T2-weighted turbo spin echo images (third row). The early T1-weighted image with fat suppression after contrast agent application (fourth row) shows a noncircumscribed mass lesion with distinct and heterogeneous enhancement and plateau curve type. The lesion was assigned a Kaiser Score^57^ of 5 and a BI-RADS 4 category and subsequently underwent ultrasound-guided biopsy, and was diagnosed as tubular adenoma. The targeted ultrasound examination (bottom row) showed a hypoechoic noncircumscribed mass corresponding to the MRI. Ultrasound guided 14G-core biopsy revealed invasive breast cancer. All MR images were acquired covering both breasts, for display purposes only the left breast with the lesion is shown. Images with the “16-channel Sentinelle Breast Coil” (Ref1) were acquired after biopsy and placement of a localization clip.

Supine breast MRI with the BraCoil was used for further assessment in a 26-year old woman who presented with a suspicious nodule detected on an MRI performed in an external facility in prone position. Magnetic resonance imaging showed an oval mass with noncircumscribed margins in the prepectoral area of the upper outer quadrant. At targeted US performed after the first prone MRI, no definite correlate was detected. Because of the matching breast geometry between the second supine MRI examination with the BraCoil and US displayed in Figure 8, the lesion could be identified and biopsied in second-look US.

As a reference, in Figure 8, the lesion location (postbiopsy marked by a clip) is shown in prone images acquired with Ref1. It can be observed that both breast shape and lesion location differ between prone reference coil and supine BraCoil images.

## DISCUSSION

We developed and successfully tested a wearable coil array enabling both supine and prone breast MRI at 3 T. With its low weight and wearability, the BraCoil is designed for strongly improved coil handling and patient comfort. A clinical case of tumor assessment with the BraCoil facilitated lesion localization compared with the standard breast coil.

The coil covers a large range of breast sizes at least up to a tested size of 90D and is expected to cover up to 90E/95D/100C based on its sensitive imaging area. Future iterations of the BraCoil will focus on increasing the SNR in the axillary and clavicular lymph nodes.

An up to 3-fold SNR increase compared with a clinical standard breast coil was demonstrated in vivo and thereby confirms the SNR gain measured on phantom in preliminary studies.^58^ In our analysis of SNR in the breast tissue, we present the data in dependence on breast volume as a measure of breast size, since it can be easily objectified. However, for such a close-fitting array of relatively small loops, the SNR more precisely depends on the effective thickness of the breast tissue between coil and chest wall, since the distance to the coil is the dominating factor for RF sensitivity. Breasts with equal volumes but very different shapes will lead to different breast tissue thickness, and, therefore, different SNR. Also, the deformability of the breast influences SNR values when comparing data from the same breast in different shapes as encountered with the different coils and prone or supine positioning. Although an effort was made to minimize contrast between water and fat tissue for the SNR measurements, the fat fraction has an impact on the measured SNR. All these factors are difficult to quantify and are regarded as natural intersubject variation explaining variations in SNR despite similar breast volumes in Figure 4.

The SNR homogeneity is similar to the clinical standard coil. As expected, gradients in signal intensity were observed due to the proximity of the coil elements to the tissue. In addition to built-in methods (prescan normalize) on the MR scanner console, robust techniques for intensity correction^59^ could be applied to provide the radiologist with more homogeneous images.

The BraCoil enables acceleration factors of up to 6 × 4 with *g*-factors below 2, which will be advantageous to accelerate the current clinical imaging protocol while conserving high SNR. Alternatively, one could keep standard examination times but add highly specific pulse sequences, for example, high-resolution DWI, which are currently often left out due to long scan times and the related high examination cost. Further, simultaneous multislice imaging techniques, particularly in 2D imaging sequences such as T2-weighted imaging and DWI, would benefit from the BraCoil's low *g*-factors in both left-right and head-foot direction. Therefore, pulse sequence protocols tailored to the strengths of the BraCoil will need to be further developed to exploit its full potential.

The supine positioning enabled by the BraCoil can bring significant advantages regarding patient comfort, coil usability, and similarity of the breast shape to other modalities. A quantitative evaluation of patient comfort by means of a questionnaire after breast MRI examinations using the clinical breast coil and the BraCoil is part of an ongoing study. To date, the sample size of study participants is still too low to report conclusions. However, qualitative feedback received by all volunteers in our study revealed that they subjectively preferred the examination using the novel flexible coil in supine (or even in prone) position over standard clinical prone breast MRI. Further, in future investigations, feedback from technologists preparing the MRI setup and executing the examination will be collected. Preliminary data suggest that preparation times with the BraCoil are shorter than with the clinical reference coil and the setup can easily be performed with a single operator.

As highlighted by the clinical showcase, supine acquisition with the BraCoil can improve lesion localization and facilitate second look US and US-guided biopsy, and, hence, reduce the number of costly MR-guided biopsies. However, lying on the back entails challenges such as motion of the patient's chest due to breathing. In addition, while the positioning is similar to breast US or the surgical table and, thus, would improve translation of imaging findings into targeted biopsies and precise surgery, the breast is less extended so that the exact localization of lesions and their relation with anatomical structures such as the nipple line may be more difficult to assess. Whether the supine position is more susceptible to gross motion of the breasts and would, for example, make follow-up examinations or the assessment of imaging features (lesion margins and heterogeneity) more difficult is subject to future investigation. Although breathing induced motion artifacts can deteriorate image quality,^60^ in the presented study, it was of little concern in healthy volunteers, since they were instructed to perform abdominal breathing to minimize chest movement. However, motion issues clearly need to be addressed in patients, as they are in a much more stressful situation. A potential solution to enable free breathing examinations would be expiratory gating with navigators^61^ or a respiratory detection device, combined with phase-encode reordering,^62^ which, however, comes at the cost of longer acquisition times. A promising alternative could be to equip the coil with on-board motion sensors^63,64^ or other motion-sensitive technology^65^ combined with online retrospective motion correction techniques.^66^

The prone position with the BraCoil yields very similar SNR compared with the supine measurement and avoids motion issues since the breasts are not moving with respiration in that case. The breast shape in this setup (Fig. 3) can be improved in future implementations by providing size-adapted curved supports on the patient table to mimic the breast shape in the supine position.

The proposed panoramic breast view reduced the number of slices to be read by a factor of 2–4 compared with the Cartesian view. Currently, it still requires manual interaction for drawing 2 curves across the breast and along the sternum, which should be automated in the future. Also, since the flattening of the breast is based only on these 2 lines, the flattening algorithm becomes less accurate toward the corners of the panoramic image. This shortcoming could be addressed by reformatting the images along a 3D curved manifold following the shape of the chest wall as suggested in reference.^67^ The technical refinement of the panoramic viewing technique is an open task, which can be addressed in the near future. Also, it is not obvious that a reduction in the number of slices with a panoramic view directly leads to shortened image interpretation times. Therefore, in future studies, we will monitor reading times of radiologists with different levels of expertise and collect their feedback regarding image quality ratings and general acceptance of the panoramic breast view in practice. Other aspects that will require a long-term investigation in the future are repeatability studies and the evaluation of the novel breast MRI technique in suitable larger patient cohorts, for example, for general breast assessment, surgery planning, or breast cancer screening.

Panoramic breast MRI together with dedicated visualization tools for diagnosis, radiation therapy and surgery planning, and artificial intelligence for lesion detection and classification, could achieve a level of diagnostic performance that would enable contrast-agent free breast examinations for assessment^68^ and MR-based screening.^45,69^

Although the clinical impact of the presented approach is yet to be demonstrated and is subject of ongoing research, we envision that panoramic breast MRI will improve cost-effectiveness, simplify the translation of diagnostic information from imaging into clinical decision making for a more personalized treatment, and will ultimately improve patient outcomes.

## Supplementary Material
