# Supplementary material for: Panoramic Magnetic Resonance Imaging of the Breast With a Wearable Coil Vest
Source: Invest Radiol. 2023 May 27;58(11):799–810. doi: 10.1097/RLI.0000000000000991 (PMC10581436; doi:10.1097/RLI.0000000000000991)

Supplemental Digital Content 1: Schematics of a single coaxial coil element.

The interface contains tuning (L_T) and matching (C_M) components, a lattice balun (C_B, L_B) and a capacitor (C_P) to obtain a phase shift for preamplifier decoupling. PIN diodes are connected via RF-blocking chokes for detuning. During transmission with the body coil the diodes are forward biased and become short-circuited. Thereby, the inner and outer conductor are shorted and the coil resonance is spoiled. A fuse is implemented in the outer conductor as a safety measure, which blows in case of too high current flow and destroys the resonance. A small preamplifier is directly connected to the interface board.

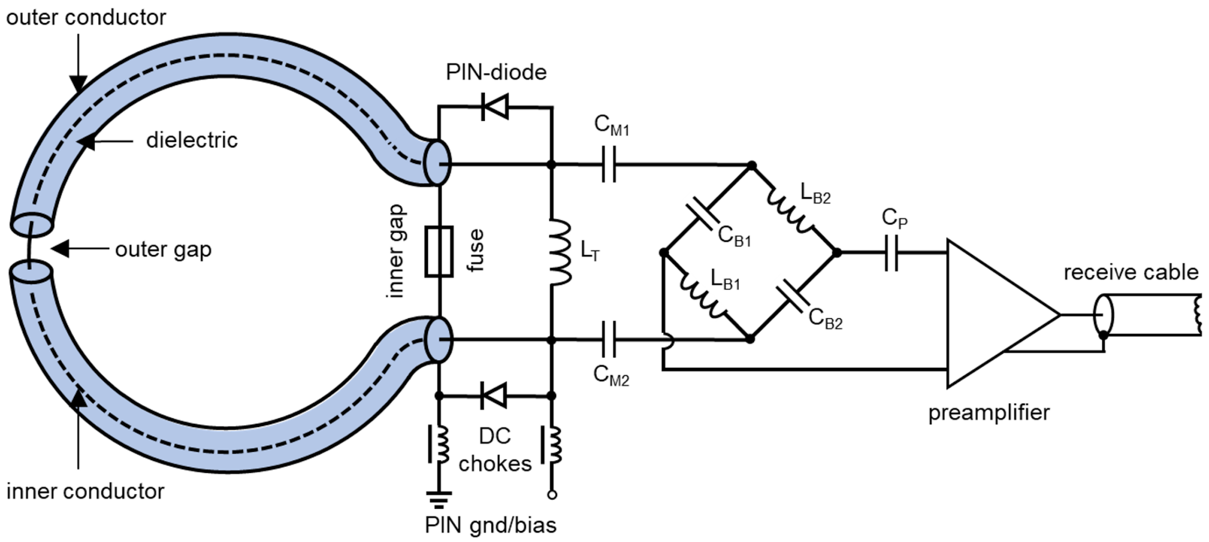

Supplement: Supplementary file 1 [file ir-58-799-s001.pdf]
