# Supplementary material for: Panoramic Magnetic Resonance Imaging of the Breast With a Wearable Coil Vest
Source: Invest Radiol. 2023 May 27;58(11):799–810. doi: 10.1097/RLI.0000000000000991 (PMC10581436; doi:10.1097/RLI.0000000000000991)
Supplement: Supplementary file 2 [file ir-58-799-s002.pdf]

Supplemental Digital Content 2

RF coil interfacing, modules and array assembly

The BraCoil is composed of 28 single-gap coaxial coils with 8 cm diameter made from thin coaxial cable (Molex 047SC-2901, Lisle, Illinois USA). The coils operate in receive-only mode at the Larmor frequency of 123.2 MHz which approximately corresponds to their self-resonance frequency. With respect to their electromagnetic behavior, it is important to distinguish between coaxial coils operated at their self-resonance and those operated off their self-resonance. The current on the outer surface of the outer conductor of the coaxial cable is responsible for the B_1 field which interacts with the sample in the MR scanner. Operated at self-resonance, the coaxial coils’ current density is homogeneous along the outer conductor. By shifting the operation frequency away from the intrinsic self-resonance of the coaxial coil, the current density distribution on the outer surface is altered, i.e., gets inhomogeneous, which affects the coils’ electromagnetic behavior[1,2].

Each coil element is individually connected to its own interface (see Figure, Supplemental Digital Content 1). Each coil interface is composed of an inductor for tuning, two capacitors for matching and a lattice LC balun (two inductors, two capacitors). Small capacitors (Exxelia, CHB series, Pessac, France) and hand-wound, air-core toroid inductors with low magnetic flux leakage are used to minimize the component footprint on the in-house designed printed circuit board. A miniaturized low noise, low input impedance preamplifier (Microwave Technology, MSM-123281, California, USA) is located on-coil, i.e., directly connected to the RF output on the coil interface, to optimize the signal-to-noise ratio.

During RF transmission with the body coil the receive-only coil is actively detuned by PIN diodes (MACOM, MA4P1250NM-1072T, Massachusetts, USA) connecting inner and outer conductor, as described by Zhang et al.[3]. RF chokes (Coilcraft, 1812CS-562XJE, Cary, Illinois, USA) block the RF signal from entering the DC feed. As an additional safety measure, a fuse is connected in the outer conductor at the location of the port, as this is

where the maximum current is expected[2]. In case of a malfunctioning of the active detuning network, the fuse is opened, thereby rendering the coil non-resonant.
“Reverse” preamplifier decoupling[3] is implemented to reduce mutual coupling between coil elements. The corresponding phase shift is realized by the balun (180°) in combination with a series capacitor. It is crucial to choose the electrical components at the balanced side of the coil’s electrical circuit to be symmetric, i.e., $C_{M1} = C_{M2}$. Asymmetric component values result in strongly decreased SNR performance of the coil[4]. The optimal overlap for coaxial coils operated on their self-resonance is similar to that of conventional coils[3] with a distance of 6 cm between coil centers. For the purpose of a form-fitted breast coil array, coaxial cable coils were deemed to be well suited, since they are very flexible and easy to manufacture, active transmission decoupling works over a broad frequency range, and they are electrically robust against bending[2].
Coaxial coils were grouped into 4-channel modules with a common interface board consisting of two stacked round double-sided printed circuit boards. All electrical components are located in the middle of the module and covered by a three-piece 3D-printed housing (see main manuscript, Fig. 1c,d). All 3D-printed parts are laser-sintered and painted PA2200. Active detuning is realized with one twisted wire pair for bias and ground per module, switching all 8 PIN diodes of the module simultaneously.
Seven of these modules are combined to form the BraCoil as shown in Fig. 1b. Three cable bundles are routed towards the right shoulder interface box and four towards the left box, all of them with a cable length below 23 cm, which is $< \lambda/4$. To suppress common mode currents on the cable shields, a floating cable trap[5] is located inside each shoulder interface box.
The BraCoil has four system cables (TIM 3G, Siemens Healthineers, Erlangen, Germany) which are fixed with a clamping mechanism inside the shoulder interface box to provide cable strain relief. To connect the cables to the newer vendor hardware version of the sockets at the patient table, a dock connector (Multi-Channel Interface 3T #11134266,

Siemens Healthineers, Erlangen, Germany) and four adapters (TIM Coil Interface 3T #10500088, Siemens Healthineers, Erlangen, Germany) are necessary.

The shape of the cover textile was designed in several iterations with the goal of fitting a large range of subject sizes taking into account in particular the overall dimensions, the position and curvature of the axilla parts, and orientation and positioning of the fixation straps. The whole array is covered and kept together by a medical grade synthetic leather (KL1100001, StoffPalette, Donaueschingen, Germany) shown in Fig. 1a (blue textile). Inside, two layers of cushion material (TG1019250, StoffPalette, Donaueschingen, Germany) as presented in Fig. 1c,d (red textile) protect the coil conductors and cabling. Each 3D-printed module housing consists of a bottom part with a flat surface on the patient side and a thread on the other side that is pushed through dedicated openings in the synthetic leather and cushion layers, thereby determining the position of each module. Coil elements were attached to the bottom cushion layer using small zip ties. The cabling from each module to the shoulder interfaces was routed above the top cushion layer to maximize distance to the subject. Above the top cover layer, a clamping ring (Fig. 1d, arrow 7) is placed that can move up and down along the threaded shaft of the bottom part without rotation. A cap (Fig. 1d, arrow 6) with a corresponding thread is then screwed onto the bottom part, thereby the ring is moved towards the bottom part and clamps the textiles together. To fit the coil to the subject, two adjustable shoulder straps (Fig. 1a, arrow 1) and an adjustable waist strap (Fig. 1a, arrow 2) are mounted on the cover layer. For maintenance, the coil can be completely reopened by unscrewing all housing caps and opening a semi-circular plastic zipper in the top cover layer (Fig. 1a, arrow 5).

References

1. Ruytenberg, T., Webb, A. & Zivkovic, I. Shielded-coaxial-cable coils as receive and transceive array elements for 7T human MRI. *Magn. Reson. Med.* **83**, 1135–1146 (2020).
2. Nohava, L. *et al.* Flexible Multi-Turn Multi-Gap Coaxial RF Coils: Design Concept and

Implementation for Magnetic Resonance Imaging at 3 and 7 Tesla. *IEEE Trans. Med. Imaging* **40**, 1267–1278 (2021).

3. Zhang, B., Sodickson, D. K. & Cloos, M. A. A high-impedance detector-array glove for magnetic resonance imaging of the hand. *Nature Biomedical Engineering* **2**, 570–577 (2018).
4. Obermann, M. *et al.* Optimization and miniaturization of Rx-only coaxial coil interfacing. in *Proc. Intl. Soc. Mag. Reson. Med.* ***28,*** 4042 (2020).
5. Seeber, D. A., Jevtic, J. & Menon, A. Floating shield current suppression trap. *Concepts Magn. Reson.* **21B**, 26–31 (2004).
