# Supplementary material for: Panoramic Magnetic Resonance Imaging of the Breast With a Wearable Coil Vest
Source: Invest Radiol. 2023 May 27;58(11):799–810. doi: 10.1097/RLI.0000000000000991 (PMC10581436; doi:10.1097/RLI.0000000000000991)
Supplement: Supplementary file 3 [file ir-58-799-s003.pdf]

Supplemental Digital Content 3

Bench and phantom MR measurements

On the bench, the BraCoil was electromagnetically characterized in terms of quality (Q)-factors, resonance frequency, impedance matching, inter-element coupling and active detuning and preamplifier decoupling performance. All measurements were carried out measuring the scattering (S-)parameter matrix on a network analyzer (E5071C, Keysight Technologies, Santa Rosa, USA).

Q-factors were measured for a single coaxial coil with a double-loop probe[1] unloaded (Q_U) and loaded (Q_L) by a 25 L gel-filled torso phantom filled with acrylic gel (σ = 0.60 S/m, ε_r = 62). The coil was loaded by the phantom, tuned once to the Larmor frequency of 123.2 MHz and $Q_U \approx 90$ and $Q_L \approx 30$ were measured, demonstrating sample noise dominance.

The S-parameter matrix containing matching (S_{ii}) and inter-element coupling (S_{ij}), was measured with the BraCoil placed on three different subjects within the study cohort (see Figure, Supplemental Digital Content 4): the smallest, a medium-sized, and the largest subject with bra sizes 70A, 85B and 90D, respectively. The preamplifiers were bridged and the coil was plugged into a self-built test rig connected to the network analyzer. The worst case matching values were -10.0 dB, -10.1 dB, and -10.0 dB, and worst case coupling was -12.4 dB, -11.7 dB, and -12.3 dB, for the small, medium and large subject, respectively.

Active detuning efficiency was evaluated via two double-loop probe measurements (S_{21}) with the coil positioned on the medium subject, bridged preamplifiers, and 50 Ω-terminated cable ends. The double-loop probe was fixed above the coil element under test and the position of probe and coil was kept constant for measurements of the same element. All coil elements were actively detuned (PIN diodes forward biased) and S_{21} was measured at the Larmor frequency. Then, the coil element under test was tuned (PIN diodes reverse biased) and the second measurement taken. This procedure was repeated for all channels and the difference (ΔS_{21}) was calculated. The resulting average ΔS_{21} = 28.9 dB, and a worst case ΔS_{21} of 21 dB proves detuning efficiency. In the same setting except the preamps not being

bridged, the performance of the preamplifier decoupling circuitry was evaluated by two S_{21} measurements with tuned coils. For the first measurement, a preamplifier was plugged and powered (10 V, 25 mA) at the RF output of the coil element under test, whereas for the second measurement the preamplifier was unplugged and the coil terminated by 50 Ω. The difference between both measurements was calculated and resulted in an average ΔS_{21} of 12.6 dB and worst case ΔS_{21} of 10.1 dB.

Tests in the MR scanner were performed with the BraCoil on the torso phantom prior to in vivo measurements. Flip-angle maps with and without the BraCoil present were acquired employing the satTFL method[2] with the scanner-integrated body coil. The relative flip-angle ratio between the two runs was calculated, showing less than 8% deviation, confirming that the implemented active detuning network and cable traps work efficiently. Furthermore, with a single coaxial coil[3], the proper functioning and correct placement of the fuse on the coaxial coil was tested. It was demonstrated that the fuse located at the outer gap worked as intended when the active detuning circuit was intentionally disabled. The fuse blew due the high current flow induced by the body coil transmission and, therefore, the coil was detuned by creating an open circuit at the outer gap, as evidenced by flip angle measurements as above. Heating of the coil was measured using fiber-optic temperature sensors (OmniFlex System, Neoptix, Canada) and pulse sequences with high specific absorption rate and fast switching of the PIN diodes. In all cases, the temperature remained below the limits indicated in ISO 60601-2-33.

References

1. Darrasse, L. & Kassab, G. Quick measurement of NMR-coil sensitivity with a dual-loop probe. *Rev. Sci. Instrum.* **64**, 1841–1844 (1993).
2. Chung, S., Kim, D., Breton, E. & Axel, L. Rapid B1+ mapping using a preconditioning RF pulse with TurboFLASH readout. *Magn. Reson. Med.* **64**, 439–446 (2010).
3. Obermann, M. *et al.* Optimization and miniaturization of Rx-only coaxial coil interfacing. in *Proc. Intl. Soc. Mag. Reson. Med. 28* 4042 (2020).
