# Supplementary material for: Panoramic Magnetic Resonance Imaging of the Breast With a Wearable Coil Vest
Source: Invest Radiol. 2023 May 27;58(11):799–810. doi: 10.1097/RLI.0000000000000991 (PMC10581436; doi:10.1097/RLI.0000000000000991)
Supplement: Supplementary file 4 [file ir-58-799-s004.pdf]

Supplemental Digital Content 4: Overview of MR pulse sequence parameters.
The pulse sequence parameters used in the study are summarized. Value ranges indicated different settings depending on breast size.

	Technical protocol			Clinical protocol							
	**Coverage assessment**	**SNR comparison**	**g-factor calculation**								
**coil (subject positioning)**	BraCoil (supine)	BraCoil (supine + prone), Body 18 (supine + prone), Breast 16 (prone)	BraCoil (prone)	BraCoil (supine)				Breast 16 (prone)			
**sequence type**	T1w 3D FLASH	T1w 3D GRE	T1w 2D GRE	T1w 3D FLASH		T2w 2D TSE	DWI 2D RESOLVE b = 0/800 s/mm^2 b_{calc} = 1400 s/mm^2	T1w 3D FLASH		T2w 2D TSE	DWI 2D EPI b = 0/800 s/mm^2 b_{calc} = 1400 s/mm^2
				high resolution	fast dynamic			high resolution	fast dynamic		
**subjects**	volunteers	volunteers	volunteers	volunteers	patient	volunteers + patient	patient	volunteers	patient	volunteers (v) + patient (p)	patient
**imaging plane**	axial	coronal	coronal	axial	coronal			axial			
**in-plane resolution [mm²]**	1.0 x 1.0	2.2 x 2.2	2.2 x 2.2	0.5 x 0.5 – 0.6 x 0.6	0.85 x 0.85	0.5 x 0.5 – 0.6 x 0.6	1.1 x 1.1	0.5 x 0.5 – 0.6 x 0.6	0.9 x 0.9	0.5 x 0.5 – 0.6 x 0.6 (v) 0.5 x 0.5 (p)	1.5 x 1.5
**field of view [mm²]**	416 x 256	224 x 422	290 x 422	152 – 192 x 346 – 384	347 x 245	240 – 280 x 332 – 350	233 x 360	152 – 192 x 380 – 384	354 x 360	200 x 345 (v) 340 x 340 (p)	178 x 340
**slice thickness [mm]**	1.3	2.2	2.5	1.0 – 1.1	1	1.5 – 1.8	1.8	1.0 – 1.1	2	1.5 – 1.8 (v) 2.5 (p)	3
**number of slices**	176	72	5	192 – 208	144	64 – 88	120	208 – 240	94	85 – 88 (v) 65 (p)	65
**phase encoding direction**	R>>L	H>>F	H>>F	R>>L	R>>L	H>>F / R>>L	H>>F	R>>L	R>>L	R>>L	H>>F
**TR/TE [ms]**	4.87 / 2.46	3.69 / 2.46	36 / 3.97	3.9 / 1.3	4.96 / 2.46	11540 –16000 / 172	13960 / 64	3.9 / 1.3	5.5 / 2.46 (TE 1) + 3.69 (TE 2)	15300 – 15910 / 172 (v) 4820 / 192 (p)	9000 / 77
**bandwidth/px. [Hz]**	400	1530	1530	390	395	145 – 150	795	390	860	148 (v) 289 (p)	1474
**flip angle [°]**	5.3	3.0	30	3	4	120	180	3	10	120	90
**acquisition time [m:ss]**	0:27	0:36	0:05	2:59 – 3:04	0:41	2:30 – 3:44	3:29	3:04	1:05	3:26 – 4:22 (v) 2:55 (p)	03:18
**acceleration factor *R***	4 x 3	1 x 1	1	2 x 1	3 x 2	3 – 4	2	2 x 1	3 x 1	3 – 4 (v) 3 (p)	2
**fat suppression**	off	off	off	SPAIR	SPAIR	off	SPAIR	SPAIR	Dixon	off	SPAIR
**sequence-specific parameters**	-	-	-	no CA	with CA, 7 post-contrast scans	turbo factor 14	5 readout shots 1 diffusion weighting	no CA	with CA, 4 post-contrast scans	turbo factor 14 (v) 21 (p)	single readout 4 diffusion weightings EPI factor 118

Abbreviations: CA… contrast agent administration, EPI… echo-planar imaging, T1/T2w… T1/T2-weighted, GRE… gradient echo, TSE… turbo spin echo, SPAIR… spectrally attenuated inversion recovery, FLASH… fast low angle shot, DWI… diffusion-weighted imaging, TR… repetition time, TE… echo time, px… pixel, R>>L… right-left direction, H>>F… head-foot direction, b_{calc}… calculated *b*-value, 2D/3D… two-/three-dimensional.
