# Supplementary material for: Panoramic Magnetic Resonance Imaging of the Breast With a Wearable Coil Vest
Source: Invest Radiol. 2023 May 27;58(11):799–810. doi: 10.1097/RLI.0000000000000991 (PMC10581436; doi:10.1097/RLI.0000000000000991)

Supplemental Digital Content 5: Scattering (S-)parameter matrix (lower triangle) and normalized noise correlation matrix (upper triangle).

S-parameters and noise-only scans were measured on three different subjects with breast volume a) 495 mL, b) 1353 mL, c) 3020 mL. The channel number (corresponding to the layout in Fig. 1) is ascending from left to right (1-28) and top to bottom (1-28). Matching is shown by the matrix diagonal (S_{ii}) and inter-element coupling (S_{ij}) is represented by the off-diagonal entries.

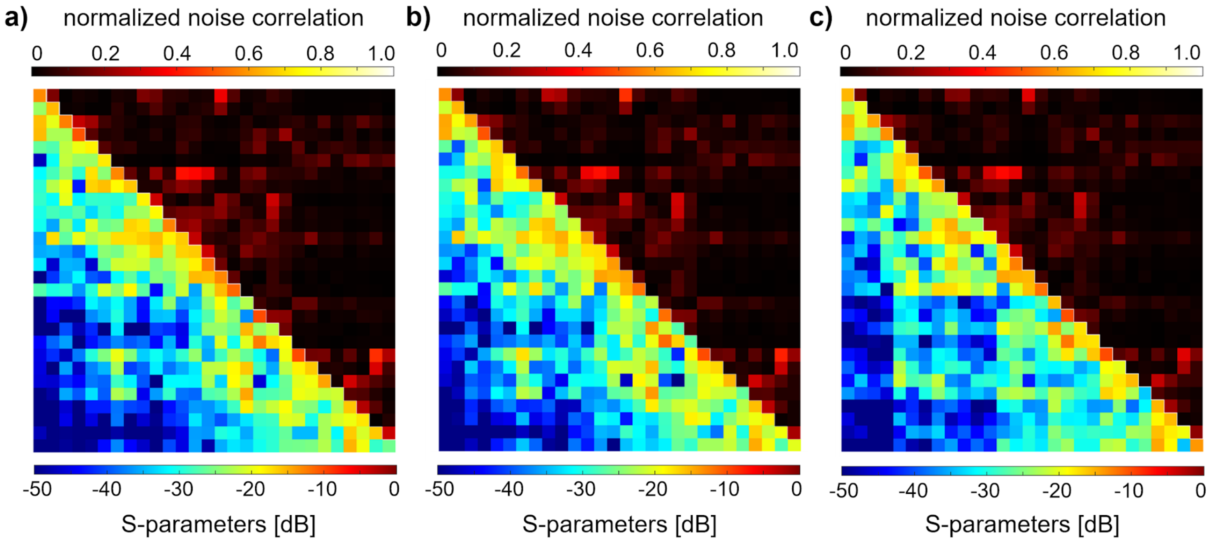

Supplement: Supplementary file 5 [file ir-58-799-s005.pdf]
