# Supplementary material for: Panoramic Magnetic Resonance Imaging of the Breast With a Wearable Coil Vest
Source: Invest Radiol. 2023 May 27;58(11):799–810. doi: 10.1097/RLI.0000000000000991 (PMC10581436; doi:10.1097/RLI.0000000000000991)

Supplemental Digital Content 6: Relative SNR homogeneity comparison between BraCoil and the reference coils.

Relative homogeneity was assessed as the standard deviation divided by the mean SNR over the segmented breast volumes. Statistically significant differences (*... $p < 0.05$, **… $p < 0.001$) are highlighted. All data points are represented by small circles. As a main result, the BraCoil's homogeneity is not significantly different from the clinical standard.The BraCoil delivers slightly more homogeneous SNR when used in supine position as compared to the prone position, which is explicable by the more even distribution of breast tissue. Due to its larger coil elements, the "Body 18" coil used in prone position achieves significantly better homogeneity than the BraCoil or the Breast 16 Sentinelle coil, at the cost of SNR. Homogeneity with the "Body 18" coil in supine position is lower than in prone position. Generally, because the coil cannot be fitted under the arms, the SNR in lateral breast tissue is considerably reduced compared to BraCoil acquisitions (see Fig. 1). Overall, the flexible coils - BraCoil and "Body 18" - show lower variability in homogeneity over different breast sizes.

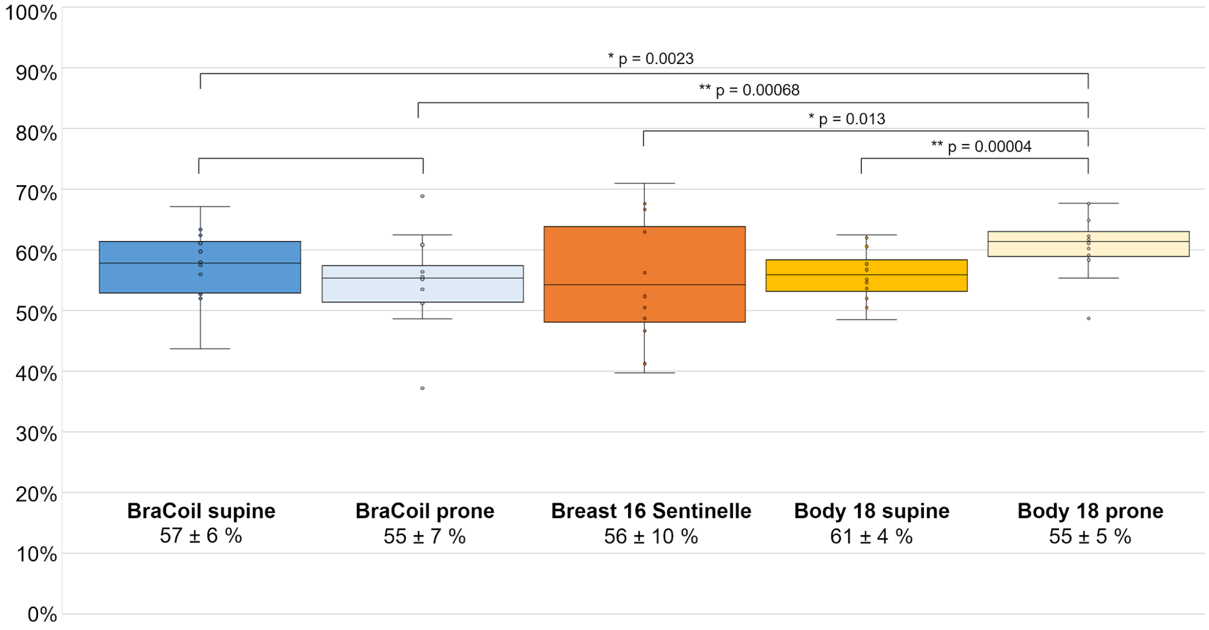

Supplement: Supplementary file 6 [file ir-58-799-s006.pdf]
