# Supplementary material for: Panoramic Magnetic Resonance Imaging of the Breast With a Wearable Coil Vest
Source: Invest Radiol. 2023 May 27;58(11):799–810. doi: 10.1097/RLI.0000000000000991 (PMC10581436; doi:10.1097/RLI.0000000000000991)

Supplemental Digital Content 7: Parallel imaging performance.

As for the medium-sized subject presented in Fig. 5, here the data for small and large breasts are shown. The distribution of the *g*-factor in a coronal slice acquired in the prone position is displayed for a) a small subject with bra size 70A and b) a large subject with bra size 90D. As for the medium-sized breasts (bra size 85B, Fig. 5), the maximum reasonably usable acceleration (g < 2) is 6 in LR-direction and 4 in HF-direction.

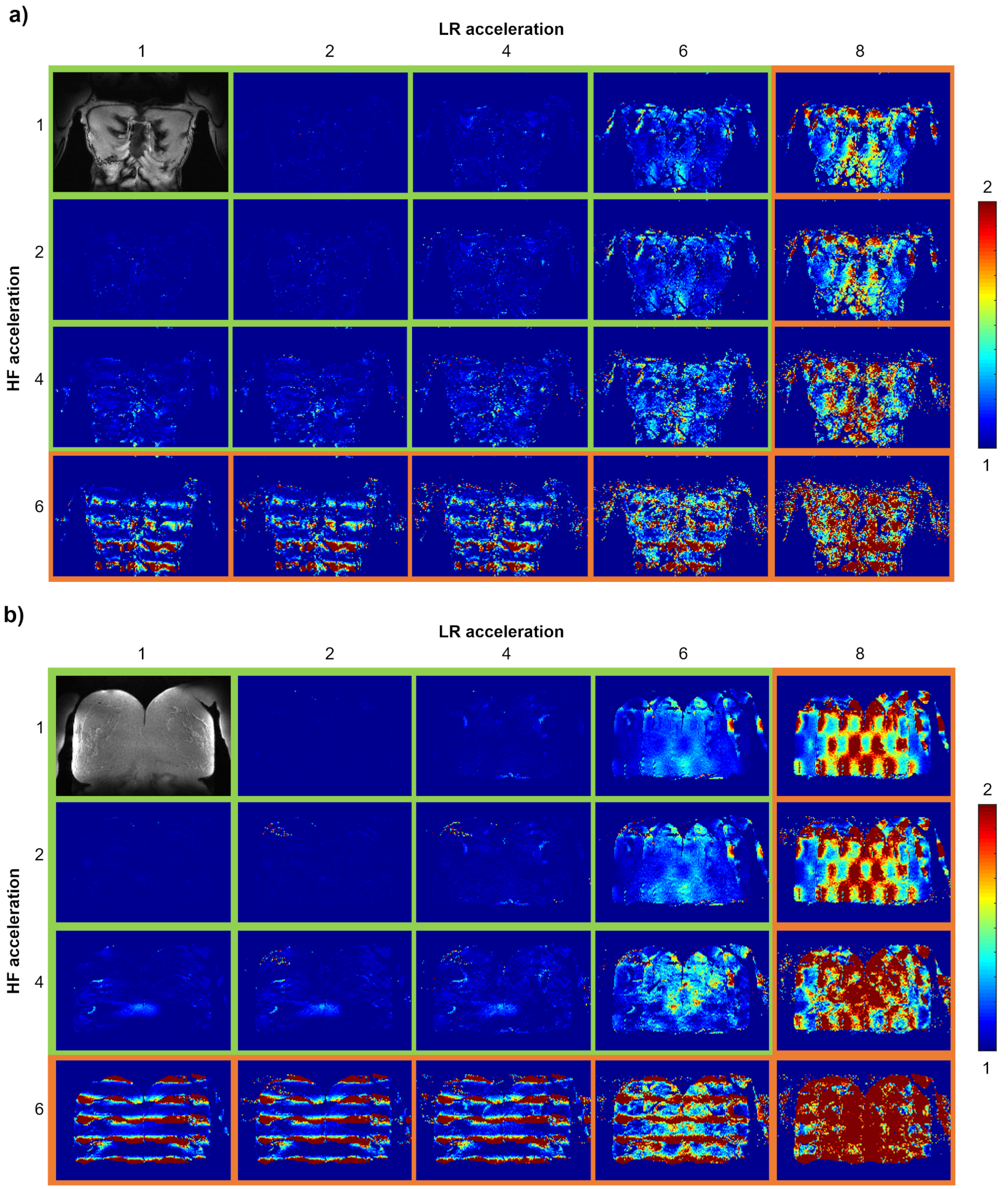

Supplement: Supplementary file 7 [file ir-58-799-s007.pdf]
