# Supplementary material for: Panoramic Magnetic Resonance Imaging of the Breast With a Wearable Coil Vest
Source: Invest Radiol. 2023 May 27;58(11):799–810. doi: 10.1097/RLI.0000000000000991 (PMC10581436; doi:10.1097/RLI.0000000000000991)
Supplement: Supplementary file 8 [file ir-58-799-s008.pdf]

Supplemental Digital Content 8

Preparation times

BraCoil “setup” comprises plugging the multichannel interface and the four TIM adapters and placing a cushion at the end of the patient table. BraCoil “positioning” includes putting the BraCoil on the subject, seated on the patient table, fine-adjusting the waist and shoulder belts, helping the subject lying down, putting on headphones, connecting the coil plugs to the adapters, and aligning the subject in the isocenter.

Reference coil Ref1 “setup” comprises placing the breast coil on the patient table, connecting the coil plugs, and putting a cushion for the feet at the end of the patient table. Reference coil Ref1 “positioning” includes helping the subject to lie on the front with the breasts hanging into the coil cups, adjusting the lateral coil elements, helping to put on headphones, and aligning the subject in the isocenter.

		BraCoil	Reference coil Ref1
**1 experienced operator**	Setup	35 ± 3 s	not possible* / 57 ± 3 s
	Positioning	97 ± 13 s	138 ± 4 s
	**total**	**132 ± 13 s**	**195 ± 5 s**
**2 experienced operators**	Setup	29 ± 5 s	50 ± 3 s
	Positioning	81 ± 2 s	117 ± 4 s
	**total**	**110 ± 5 s**	**167 ± 5 s**
**1 inexperienced** operator (runs 1 to 3)**	Setup	46 / 42 / 36 s	
	Positioning	147 / 112 / 99 s	
	total	193 / 154 / 135 s	

* … operator-dependent as the coil is large and heavy. If the risk of damaging the coil or the operator’s health is too high, only a two-operator setup can be performed.

**... the operator never handled the BraCoil before but has experience with setting up MRI head coils.
